# Differential physiological, nutritional and molecular responses to lead stress in garden balsam and ornamental kale

**DOI:** 10.1038/s41598-026-46310-6

**Published:** 2026-04-03

**Authors:** Nurselin Yilmaz, Sergul Ergin

**Affiliations:** Department of Agricultural Biotechnology, Faculty of Agriculture, Eskisehir Osmangazi University, Eskisehir, Turkey

**Keywords:** Garden balsam, Ornamental kale, Pb stress, Heat shock proteins, Phytoremediation, Proline, Environmental sciences, Physiology, Plant sciences

## Abstract

Lead is a widespread environmental pollutant with high toxicity. Ornamental plants are promising candidates for phytoremediation because they provide aesthetic value and are not directly used in human nutrition. In this study, Pb stress was induced in garden balsam and ornamental cabbage by applying 0, 100, 200, and 400 ppm Pb(NO_3_)_2_, and their physiological, biochemical, and molecular responses were examined to evaluate their tolerance and phytoremediation potential. Pb accumulation was mainly concentrated in roots, with higher levels in ornamental cabbage. Pb exposure altered nutrient uptake, although the extent of this effect varied depending on the element and plant species. Ion leakage and turgor pressure were moderately affected, but damage remained below thresholds for irreversible injury, indicating tolerance. Chlorophyll content was maintained, likely due to stable magnesium levels. Biomass was largely unaffected and slightly increased under some conditions. Proline accumulated in garden balsam at higher Pb levels but decreased in ornamental cabbage. Total soluble proteins declined in garden balsam but increased in ornamental cabbage, reflecting stronger protein homeostasis. Heat shock proteins (HSP60 and HSP23) were strongly induced in ornamental cabbage, whereas garden balsam showed limited or declining expression. Overall, ornamental cabbage demonstrated higher detoxification and stress tolerance, highlighting its greater potential for phytoremediation of Pb-contaminated environments.

**Supplementary Information:**

The online version contains supplementary material available at 10.1038/s41598-026-46310-6.

## Introduction

Soil contamination by heavy metals is a critical global environmental problem due to its persistent and toxic nature, posing long-term risks to ecosystems and human health^1^. Heavy metals enter soils through natural and anthropogenic sources and accumulate because they are non-biodegradable, forming complex and often harmful interactions within biological systems^2,3^. Among them, lead (Pb) is of particular concern due to its historical widespread use and strong toxicity^4^. According to the United States Environmental Protection Agency (EPA), Pb levels above 400 ppm in residential soils and 1200 ppm in non-residential or garden soils pose significant hazards^5^. Exceeding these thresholds severely disrupts plant morphological, physiological, and biochemical processes^6^.

Pb taken up by roots accumulates predominantly in root tissues, with limited movement to aerial organs^7^. Once inside the plant, Pb interferes with essential activities such as photosynthesis, respiration, and cellular metabolism, leading to reduced growth, restricted mineral uptake, and impaired germination^8^. A major consequence of Pb toxicity is disruption of nutrient homeostasis, particularly for ions such^9,10^ as Mn^2^⁺, Zn^2^⁺, Fe^2^⁺, Ca^2^⁺, and Mg^2^⁺. Pb is thought to compete with these ions for common transport channels, disturbing ionic balance and impairing enzymatic activity. Such nutritional imbalances further exacerbate oxidative stress and reduce metabolic efficiency in plants^7^.

Pb stress also alters plant water relations by limiting transpiration and reducing leaf area through disturbances in root-to-shoot transport^11^. Water deficit subsequently affects osmotic adjustment mechanisms, especially the accumulation of osmolytes, such as soluble sugars, amino acids, and polyamines, which play key roles in turgor maintenance and stress protection^12^. Among these, proline is one of the most widely studied osmolytes, known for its role in ROS scavenging and stabilization of proteins and membranes under stress. Numerous studies have shown that Pb exposure induces substantial proline accumulation across various plant species^13–15^.

In addition to osmotic regulation, heavy metal stress triggers significant changes in protein metabolism. Plants frequently enhance the synthesis of heat shock proteins (HSPs), which function as molecular chaperones involved in protein folding, stabilization, and protection against stress-induced denaturation^13,16^. Metal-induced upregulation of major HSP families, including HSP70, HSP90, and small HSPs, has been reported in species such as Arabidopsis, tomato, *Brassica juncea*, and soybean^17–20^. These findings suggest that HSP-mediated proteostasis, together with osmolyte accumulation, constitutes a central protective mechanism under heavy metal stress.

Growing awareness of heavy metal pollution has increased interest in phytoremediation as a sustainable and eco-friendly remediation technology^21^. Ornamental plants are particularly advantageous in this context because they do not enter the food chain and are well-suited for urban landscapes. However, the effectiveness of phytoremediation largely depends on a plant’s capacity to withstand metal toxicity through efficient nutrient management, osmotic balance, and stress-responsive protein regulation.

Despite extensive research on HSP responses in model and crop plants, information regarding their behaviour under Pb stress in ornamental species remains limited. *Impatiens balsamina* (garden balsam) and *Brassica oleracea* var. *acephala* (ornamental kale) are widely used ornamental species with potential phytoremediation capabilities, yet their physiological and molecular responses to Pb remain poorly understood.

Therefore, the present study aims to investigate the effects of Pb exposure on nutrient dynamics, osmolyte accumulation (focusing on proline) and HSP expression in two species with potential phytoremediation ability, *I. balsamina* and *B. oleracea* var. *acephala*. Elucidating their physiological responses to Pb stress will contribute to a deeper understanding of their tolerance mechanisms and inform future phytoremediation strategies.

## Results

### Pb accumulation in growth medium and in plants

**Fig. 1:**
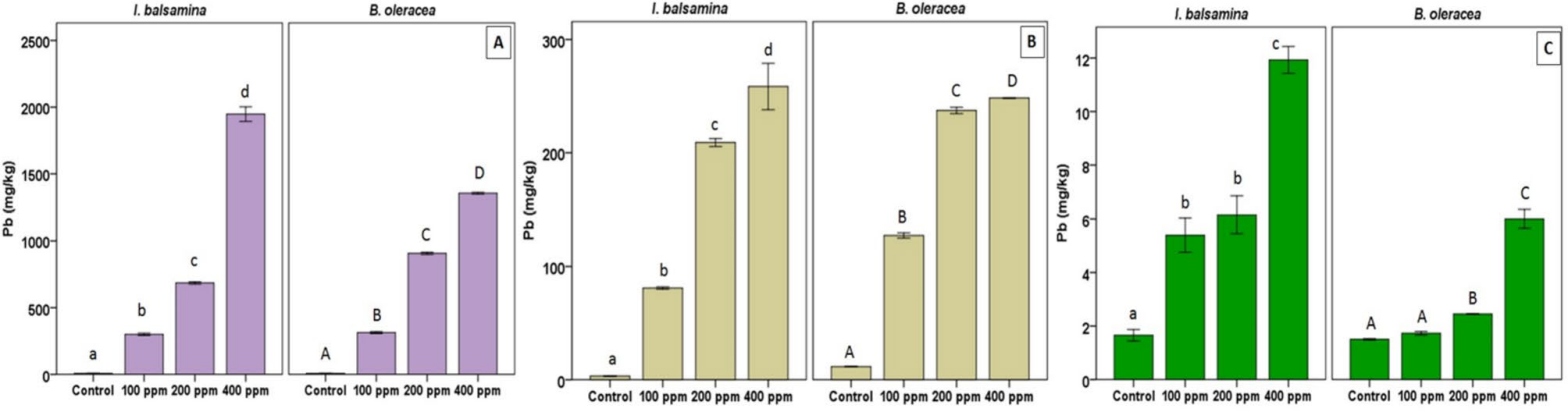
Lead accumulation in the growth medium (**A**), roots (**B**), and leaves (**C**) of *I. balsamina* and *B. oleracea var. acephala* under Pb treatments. Bars represent mean ± SD (n = 3). Lowercase letters denote significant differences within *I. balsamina*; uppercase letters denote significant differences within *B. oleracea var. acephala* (*p* < 0.05, Duncan’s test).

Lead accumulation in the growth medium, roots, and leaves of *I. balsamina* and *B. oleracea* var. *acephala* is presented in Fig. 1. Increasing external Pb concentrations led to higher Pb levels in the growth medium. In *I. balsamina*, Pb accumulation reached 8 mg kg^−1^ (control), 300 mg kg^−1^ (100 ppm), 685 mg kg^−1^ (200 ppm), and 1947 mg kg^−1^ (400 ppm). For *B. oleracea* var. *acephala*, these values were 8, 313, 907, and 1355 mg kg^-1^, respectively (Fig. 1A). The low Pb concentration detected in control plants (8 mg kg⁻^1^) represents background Pb naturally present in the growth medium or irrigation water (Table 1).

**Table 1:** Physicochemical properties of the growth medium before the treatments.

Soil property	
Potassium (K_2_O) kg/da	41.46
Phosphor (P_2_O_5_) kg/da	11.50
Lime (%)	6.84
Organic matter (%)	6.24
Total salt (dS/m)	0.80
pH	7.24
Saturation (%)	80

Root Pb levels also increased with Pb treatments. *I. balsamina* showed 3.2 mg kg^−1^ (control), 81.0 mg kg^−1^ (100 ppm), 209.0 mg kg^−1^ (200 ppm), and 258.5 mg kg^−1^ (400 ppm), while *B. oleracea* var. *acephala* had 11.6, 127.3, 237.3, and 248.3 mg kg^−1^, respectively. Root Pb accumulation was generally higher in *B. oleracea* var. *acephala*, except at 400 ppm (Fig. 1B). Similarly, foliar Pb concentrations increased with Pb exposure. *I. balsamina* accumulated 1.7, 5.4, 6.2, and 11.9 mg kg^−1^ Pb across treatments, while *B. oleracea* var. *acephala* accumulated 1.5, 1.7, 2.5, and 6.0 mg kg^−1^. Unlike the roots, foliar Pb accumulation was consistently higher in *I. balsamina* (Fig. 1C).

Bioenrichment factor (BCF) values for *B. oleracea* var. *acephala* were 1.59 (control), 0.41 (100 ppm), 0.26 (200 ppm), and 0.19 (400 ppm), compared with 0.60, 0.29, 0.31 and 0.14 in *I. balsamina*.

### Mineral nutrient concentration

The effects of Pb treatments on K, Ca, Mg, P, Cu, Fe, Mn, and Zn concentrations in roots and leaves of *I. balsamina* and *B. oleracea* var. *acephala* are summarized in Tables 2 and 3. In *I. balsamina* roots, K increased at 100 ppm and then declined at higher Pb levels. In *B. oleracea* var. *acephala*, the lowest root K occurred at 100 ppm, while the highest level was recorded at 200 ppm. Leaf K in *I. balsamina* decreased at 100 ppm, peaked at 200 ppm, and slightly increased at 400 ppm. *B. oleracea* leaves showed the lowest K in the control and higher values under all Pb treatments.

**Table 2:** Effect of Pb treatments on the content of K, Ca, Mg, P, Cu, Fe, Mn and Zn in the roots.

Species	Treatments	K (mg kg^−1^)	Ca (mg kg^−1^)	Mg (mg kg^−1^)	P (mg kg^−1^)	Cu (mg kg^−1^)	Fe (mg kg^−1^)	Mn (mg kg^−1^)	Zn (mg kg^−1^)
*I. balsamina*	Control	37,776.7 ± 1018.6 c	15,326.7 ± 891.6*	9650.3 ± 52.88 a	2932.0 ± 133.2 b	66.4 ± 3.0*	402.7 ± 32.9*	17.0 ± 0.8 a	48.9 ± 3.4 a
	100 ppm	44,613.3 ± 474.3 d	13,470.0 ± 112.8*	8076.7 ± 106.6 a	2821.3 ± 166.1 b	71.2 ± 5.9*	438.7 ± 20.9*	21.9 ± 0.2 b	54.4 ± 1.9 ab
	200 ppm	33,740.0 ± 1944.5 b	14,410.0 ± 485.6*	11,991.0 ± 641.7 b	2212.3 ± 60.0 a	77.5 ± 1.2*	379.3 ± 44.2*	18.0 ± 0.9 ab	53.2 ± 2.8 ab
	400 ppm	26,093.3 ± 696.1 a	15,200.0 ± 320.7*	12,940.3 ± 1488.9 b	1964.3 ± 42.0 a	75.6 ± 0.9*	434.0 ± 60.7*	21.1 ± 2.2 ab	63.7 ± 4.0 b
*B. oleracea* var. *acephala*	Control	23,953.3 ± 515.3 B	7140.3 ± 940.3 A	4847.7 ± 110.6*	7691.3 ± 456.6 C	82.5 ± 1.0 B	2270.5 ± 127.0 D	39.5 ± 3.2 B	93.3 ± 3.7 B
	100 ppm	20,883.3 ± 672.7 A	9970.0 ± 407.2 B	4568.0 ± 317.9*	5852.0 ± 275.5 B	80.9 ± 0.3 B	1097.0 ± 20.5 C	29.6 ± 0.4 A	99.3 ± 5.0 B
	200 ppm	29,496.7 ± 789.5 C	10,798.3 ± 636.9 B	4414.7 ± 91.1*	5123.7 ± 198.9 AB	84.6 ± 1.3 B	632.7 ± 42.2 B	31.2 ± 2.2 AB	71.1 ± 1.6 A
	400 ppm	28,590.0 ± 601.2 C	8947.3 ± 1011.5 AB	4643.0 ± 90.9*	4554.7 ± 219.7 A	69.2 ± 1.6 A	423.7 ± 49.5 A	31.8 ± 3.8 AB	80.3 ± 0.7 A

Significant differences between treatments are shown in lower case letters (a, b and c) in *I. balsamina* and in upper case letters (A, B and C) in *Brassica oleracea* var. *acephala* (*p* < 0.05), *non-significant. Results are the means of three replicates ± SD.

**Table 3:** Effect of Pb treatments on the content of K, Ca, Mg, P, Cu, Fe, Mn and Zn in the leaves.

Species	Treatments	K (mg kg^−1^)	Ca (mg kg^−1^)	Mg (mg kg^−1^)	P (mg kg^−1^)	Cu (mg kg^−1^)	Fe (mg kg^−1^)	Mn (mg kg^−1^)	Zn (mg kg^−1^)
*I. balsamina*	Control	14,493.3 ± 1142.6 ab	19,753.3 ± 1275.2 a	7216.0 ± 91.0 a	5175.7 ± 115.2 c	39.7 ± 1.2 b	76.9 ± 0.2 c	28.8 ± 3.2 a	38.8 ± 0.8 c
	100 ppm	12,940.0 ± 1117.1 a	20,563.3 ± 553.2 ab	7095.3 ± 492.7 a	5473.7 ± 266.9 c	31.1 ± 0.5 a	76.8 ± 0.2 c	43.6 ± 1.2 c	33.5 ± 2.0 b
	200 ppm	16,693.3 ± 667.9 b	24,356.7 ± 1307.8 c	8088.0 ± 289.9 ab	3804.0 ± 84.5 b	34.1 ± 2.4 a	64.2 ± 0.9 a	33.7 ± 0.8 ab	29.4 ± 2.0 ab
	400 ppm	15,346.7 ± 471.1 ab	23,486.7 ± 707.7 bc	8569.0 ± 154.3 b	3128.3 ± 230.5 a	32.9 ± 0.8 a	67.3 ± 1.0 b	38.6 ± 0.7 bc	27.5 ± 0.8 a
*B. oleracea* var. *acephala*	Control	24,646.7 ± 807.7 A	22,729.0 ± 745.7 B	4931.0 ± 137.7 A	5940.7 ± 177.2 B	42.2 ± 0.2 C	50.6 ± 1.0 A	25.4 ± 0.2 A	46.6 ± 1.1 C
	100 ppm	33,410.0 ± 1073.2 B	22,676.7 ± 916.4 B	5479.3 ± 99.2 B	5268.0 ± 59.8 A	31.3 ± 0.4 A	46.8 ± 0.2 A	29.7 ± 0.7 B	40.7 ± 0.9 B
	200 ppm	32,256.7 ± 1021.9 B	22,676.7 ± 916.4 B	4995.3 ± 85.0 A	6002.7 ± 58.4 B	37.8 ± 0.4 B	51.1 ± 1.3 A	31.6 ± 1.7 B	37.9 ± 1.3 AB
	400 ppm	33,140.0 ± 1472.2 B	19,436.7 ± 514.1 A	5656.7 ± 157.8 B	5105.3 ± 232.2 A	32.3 ± 0.1 A	56.9 ± 2.0 B	32.6 ± 1.0 B	35.3 ± 2.2 A

Significant differences between treatments are shown in lower case letters (a, b and c) in *I. balsamina* and in upper case letters (A, B and C) in *Brassica oleracea* var. *acephala* (*p* < 0.05), *non-significant. Results are the means of three replicates ± SD.

Root Ca in *B. oleracea* var. *acephala* increased up to 200 ppm and declined at 400 ppm, while *I. balsamina* roots showed no significant changes. In leaves, Ca increased in *I. balsamina* across all Pb treatments, whereas *B. oleracea* var. *acephala* showed similar Ca levels at 0–200 ppm but a decrease at 400 ppm.

Root Mg in *I. balsamina* increased at 200 and 400 ppm, while *B. oleracea* var. *acephala* showed no significant changes. Leaf Mg increased in both species at higher Pb concentrations, though the rise was more evident in *I. balsamina*.

In *I. balsamina* roots, P remained similar to the control at 100 ppm but decreased at 200 and 400 ppm. *B. oleracea* var. *acephala* roots showed a consistent decline with increasing Pb. In leaves, P decreased at higher Pb levels in *I. balsamina*, whereas in *B. oleracea* var. *acephala*, P was stable at 200 ppm but lower at 100 and 400 ppm.

The amount of Cu in the roots of *I. balsamina* was unaffected by Pb. In *B. oleracea* var. *acephala*, Cu remained unchanged up to 200 ppm but decreased at 400 ppm. In leaves of both species, Cu concentrations declined relative to the control.

While Fe content in *I. balsamina* roots did not show a significant response to Pb, a dose-dependent decrease was observed in *B. oleracea* var. *acephala* roots. Fe content in *I. balsamina* leaves decreased at 200 ppm with a slight recovery at 400 ppm. In *B. oleracea* var. *acephala* leaves, Fe remained unchanged up to 200 ppm and increased at 400 ppm.

*I. balsamina* roots showed increased Mn under Pb, especially at 100 ppm. *B. oleracea* var. *acephala* roots exhibited decreased Mn, with the lowest value at 100 ppm. Leaf Mn increased in *I. balsamina*, while *B. oleracea* var. *acephala* showed higher Mn than the control but no differences among treatments.

In *I. balsamina* roots, Zn slightly increased at 100–200 ppm and reached the highest level at 400 ppm. In *B. oleracea* var. *acephala*, root Zn remained stable at 100 ppm but declined at 200–400 ppm. Leaf Zn decreased progressively with increasing Pb in both species.

### Physiological parameters

The effects of Pb treatments on injury, RWC, turgor loss, and chlorophyll content are summarized in Table 4. Injury in both species increased proportionally with rising Pb concentrations. While RWC remained unchanged, turgor loss rose from 9.1 to 19.9% in *I. balsamina* and from 8.07 to 12.13% in *B. oleracea* var. *acephala*. Chlorophyll content was unaffected by Pb, remaining stable at 2.6–2.8 mg gFW^-1^ in *I. balsamina* and 2.3–2.4 mg gFW^-1^ in *B. oleracea* var. *acephala*.

**Table 4:** Effect of Pb treatments on the injury, RWC, loss of turgor and chlorophyll content in the leaves.

Species	Treatments	Injury (%)	RWC (%)	Loss of turgor (%)	Chlorophyll (mg gFW^−1^)
*I. balsamina*	Control		82.2 ± 4.0*	9.3 ± 0.8 a	2.6 ± 0.1*
	100 ppm	1.8 ± 0.1 a	85.0 ± 2.6*	12.3 ± 0.6 b	2.7 ± 0.1*
	200 ppm	3.6 ± 0.2 b	85.6 ± 2.2*	12.9 ± 1.1 b	2.8 ± 0.1*
	400 ppm	6.0 ± 0.3 c	78.1 ± 3.5*	19.9 ± 1.6 c	2.8 ± 0.2*
*B. oleracea* var. *acephala*	Control		89.8 ± 3.0*	8.1 ± 0.7 A	2.4 ± 0.2*
	100 ppm	1.6 ± 0.1 A	91.5 ± 5.6*	9.8 ± 0.6 B	2.4 ± 0.0*
	200 ppm	2.5 ± 0.1 B	88.5 ± 1.9*	10.6 ± 0.6 B	2.4 ± 0.1*
	400 ppm	3.6 ± 0.2 C	86.9 ± 2.1*	12.1 ± 0.6 C	2.3 ± 0.0*

Significant differences between treatments are shown in lower case letters (a, b and c) in *I. balsamina* and in upper case letters (A, B and C) in *B. oleracea* var. *acephala* (*p* < 0.05), *non-significant. Results are the means of three replicates ± SD.

### Plant biomass

In *I. balsamina*, root fresh weight increased at 200 and 400 ppm, whereas a reduction occurred at 100 ppm. In *B. oleracea* var. *acephala,* it remained unchanged at 100 ppm but decreased at 200 and 400 ppm. Shoot fresh weight in *I. balsamina* decreased at 100 and 200 ppm but increased at 400 ppm. In *B. oleracea* var. *acephala*, shoot fresh weight increased at all Pb concentrations except 200 ppm. In *I. balsamina*, the highest root dry weight was observed in the control. Although slight increases occurred with rising Pb concentration, overall reductions were still evident. In *B. oleracea* var. *acephala*, root dry weight increased only at 100 ppm. Shoot dry weight remained unchanged in *I. balsamina*. In contrast, *B. oleracea* var. *acephala* exhibited slight increases, particularly at 100 and 400 ppm (Table 5).

**Table 5:** Effect of Pb treatments on the root and shoot FW and root and shoot DW of species.

Species	Treatments	FW (g)		DW (g)	
		Root	Shoot	Root	Shoot
*I. balsamina*	Control	15.35 ± 0.6 b	48.55 ± 1.3 ab	0.81 ± 0.0 a	4.62 ± 0.3*
	100 ppm	12.80 ± 0.4 a	41.17 ± 0.8 c	0.57 ± 0.0 c	4.41 ± 0.5*
	200 ppm	16.16 ± 0.3 bc	43.21 ± 1.6 bc	0.68 ± 0.0 bc	3.99 ± 0.3*
	400 ppm	17.21 ± 0.1 c	54.70 ± 2.7 a	0.78 ± 0.0 ab	4.64 ± 0.4*
*B. oleracea*var. a*cephala*	Control	2.77 ± 0.1 A	31.39 ± 1.5 A	0.33 ± 0.0 A	4.07 ± 0.0 A
	100 ppm	2.90 ± 0.2 A	41.49 ± 0.6 B	0.50 ± 0.0 B	5.24 ± 0.3 B
	200 ppm	1.96 ± 0.1 B	32.67 ± 1.7 A	0.32 ± 0.0 A	4.65 ± 0.1 AB
	400 ppm	1.47 ± 0.0 B	42.26 ± 2.0 B	0.29 ± 0.0 A	5.52 ± 0.3 B

Significant differences between treatments are shown in lower case letters (a, b and c) in *I. balsamina* and in upper case letters (A, B and C) in *B. oleracea* var. *acephala* (*p* < 0.05), * non-significant. Results are the means of three replicates ± SD.

### Proline content and protein analysis

**Fig. 2:**
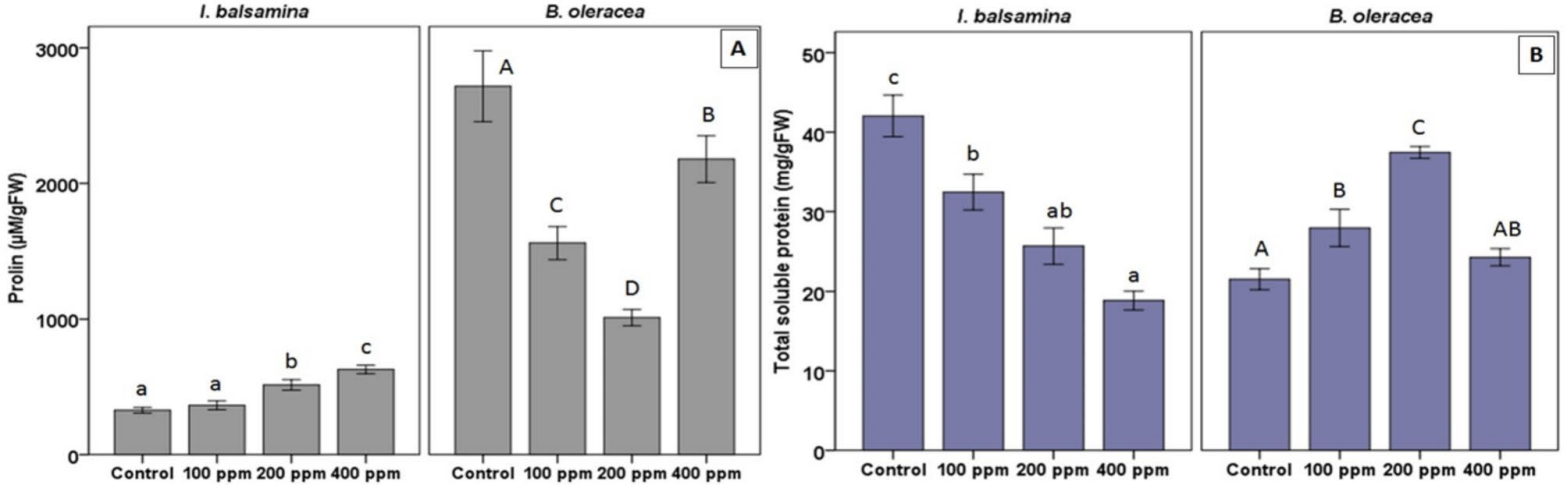
Proline content (**A**) and total soluble protein (**B**) in *I. balsamina* and *B. oleracea* var. *acephala* under Pb treatments. Bars represent mean ± SD (n = 3). Lowercase letters indicate significant differences among treatments in *I. balsamina*, and uppercase letters indicate differences in *B. oleracea* var. *acephala*. (*p* < 0.05, Duncan’s test).

In *I. balsamina*, proline content was similar to the control (327.7 µM gFW^−1^), at 100 ppm (364.1 µM gFW^−1^) but increased markedly at 200 (506.1 µM gFW^−1^) and 400 ppm (629.1 µM gFW^−1^). In *B. oleracea* var. *acephala*, it decreased at 100 (1559.6 µM gFW^−1^) and 200 ppm (1011.7 µM gFW^−1^), and rose at 400 ppm (2179.2 µM gFW^−1^), yet remained below the control level (2716.9 µM gFW^−1^) across all treatments (Fig. 2A).

Pb treatments caused a progressive decline in TSP in *I. balsamina*, decreasing from 42.04 mg gFW^−1^ (control) to 32.45, 25.66, and 18.85 mg gFW^−1^ at 100, 200, and 400 ppm, respectively (Fig. 2B). In *B. oleracea* var. *acephala*, TSP increased at 100 and 200 ppm (27.94 and 37.45 mg gFW^−1^) but declined slightly at 400 ppm (24.28 mg gFW^−1^), though remaining comparable to the control (21.53 mg gFW^−1^).

**Fig. 3:**
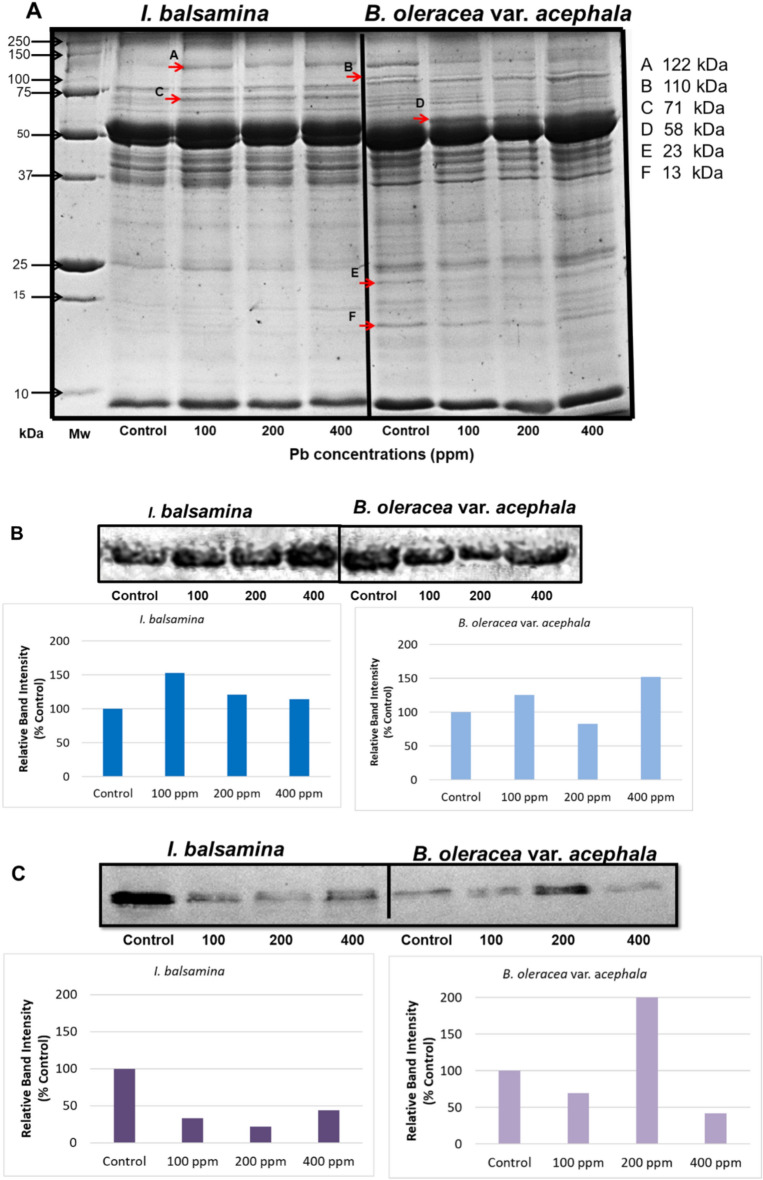
(**A**) SDS-PAGE profiles of total soluble proteins in *I. balsamina* and *B. oleracea* var. *acephala*, Pb-responsive bands are indicated by red arrows. Each lane contains 30 μg protein. (**B**) Immunoblot detection of HSP60. (**C**) Immunoblot detection of HSP23. Band intensities were quantified relative to the corresponding control (set to 100%). The blot shown is cropped for clarity. Original uncropped blot is provided in Supplementary Figures.

Figure 3A shows total protein profiles of *I. balsamina* and *B. oleracea* var. *acephala* under Pb treatments. In *I. balsamina*, Pb induced a ~ 122 kDa band absent in the control and a ~ 71 kDa band, which is not detected in *B. oleracea* var. *acephala*. In *B. oleracea* var. *acephala*, the ~ 122 kDa band decreased under Pb, and a ~ 110 kDa band appeared only at control and 400 ppm. Pb enhanced a 58 kDa band in both species, while two low molecular weight protein bands (~ 23 and ~ 13 kDa), prominently detected in *B. oleracea* var. *acephala*, appeared only faintly in *I. balsamina*.

Fig. 3B and C display the expression profiles of HSPs in both species, as determined by immunoblot analysis using antibodies specific to the respective HSPs. In *I. balsamina*, HSP60 increased with Pb, peaking at 100 ppm (153%), while HSP23 decreased with Pb, slightly rising at 400 ppm (44% of control). In *B. oleracea* var. *acephala*, HSP60 fluctuated with Pb (82–152%), and HSP23 was strongly induced at 200 ppm (207%) but decreased at 100 and 400 ppm.

## Discussion

Pb bioavailability in soils depends on physicochemical factors such as pH, organic matter content, and soil type, and background Pb levels are commonly detected even in non-contaminated substrates^22,23^. In this study, the low Pb concentrations detected in control plants (8 mg kg^−1^) fall within this background range and therefore do not indicate experimental contamination. Increasing external Pb supply resulted in a marked rise in Pb accumulation in the growth medium, confirming the effectiveness and reliability of the applied treatments.

Pb is known to exhibit low mobility in plants due to its strong binding to cell walls and sequestration in vacuoles, leading to preferential retention in roots^13,8^. Consistent with this, both species accumulated substantially higher Pb concentrations in roots than in leaves. However, clear species-specific differences were observed. In the context of phytoremediation, phytostabilization refers to the immobilization and retention of metals primarily in root tissues, thereby limiting their translocation, whereas phytoextraction involves efficient uptake and transfer of metals to aboveground parts. Based on these definitions, *B. oleracea* var. *acephala* predominantly exhibits a phytostabilization-oriented strategy through root Pb retention, while *I. balsamina* shows characteristics more consistent with a phytoextraction-oriented response due to its relatively higher Pb translocation to shoots. The observed decline in BCF values with increasing Pb concentration likely reflects saturation of uptake systems, reduced translocation efficiency, and physiological constraints associated with Pb toxicity, which collectively limit further Pb accumulation relative to soil Pb levels.

Pb stress is widely reported to disrupt macro- and micronutrient uptake, primarily by interfering with membrane transport systems and inducing ionic competition^9,24,7^. In the present study, Pb-induced alterations in nutrient profiles were species- and organ-specific. Potassium uptake was more strongly affected in *I. balsamina* roots, whereas *B. oleracea* var. *acephala* maintained relatively stable K levels, suggesting more effective ionic homeostasis. Despite frequent reports of Pb-induced Ca depletion, Ca concentrations remained largely unchanged in both species, indicating a limited impact of Pb on Ca transport under the applied conditions. Magnesium responses differed between species, with increased root Mg in *I. balsamina* but no significant change in *B. oleracea* var. *acephala*, further highlighting distinct adaptive strategies. Phosphorus was reduced in roots of both species, while leaf P declined only in *I. balsamina*, partially aligning with previous observations of Pb-induced P limitation.

Micronutrient responses also varied between species. Root Cu concentrations were largely unaffected, whereas leaf Cu declined, consistent with reports in *Vallisneria natans* under Pb stress^25^. Pb exposure reduced root Fe in *B. oleracea* var. *acephala* but not in *I. balsamina*, reflecting the inconsistent Fe responses reported across plant species^26,25^. While Mn and Zn levels declined in roots of *B. oleracea* var. *acephala*, both species exhibited increased shoot concentrations, suggesting enhanced translocation or altered redistribution under Pb stress. Such contrasting responses emphasize the strong species dependence of micronutrient regulation under heavy metal exposure.

Heavy metal stress commonly compromises membrane integrity, resulting in increased ion leakage, which serves as a reliable indicator of cellular injury^27^. Although Pb exposure induced membrane damage in both species, injury levels remained relatively low compared with those reported for more Pb-sensitive plants^28–30^. Notably, injury did not exceed the threshold associated with irreversible membrane damage, indicating a degree of inherent Pb tolerance in both species, particularly in *B. oleracea* var. *acephala*.

Pb-induced disruption of membrane function and osmotic balance often leads to turgor loss^31^. Both species exhibited reduced turgor under Pb exposure, although differences between moderate Pb treatments were not significant. This response is consistent with previous reports and reflects a common physiological adjustment to Pb-induced water relations stress^30,32^.

Pb toxicity is frequently associated with oxidative stress and chloroplast damage, which can impair chlorophyll biosynthesis by interfering with Mg availability or key enzymatic steps^33,34^. In contrast to many reports describing Pb-induced chlorophyll reduction^35,9^, total chlorophyll content remained largely unchanged in both species. The stability of leaf Mg concentrations, particularly in *I. balsamina*, likely contributed to the maintenance of chlorophyll levels. These findings support the notion that chlorophyll responses to Pb stress are highly species- and concentration-dependent, and may vary with exposure duration and Pb availability.

Plant growth responses to Pb stress are often characterized by reduced biomass at high concentrations, whereas low to moderate levels may induce hormetic effects^36–38^. In this study, shoot and root biomass were either unaffected or slightly increased, suggesting that the applied Pb concentrations fell within a hormetic range and did not impose severe growth inhibition.

Proline accumulation is a well-established adaptive response to heavy metal stress, functioning in osmotic adjustment, ROS scavenging, and metal chelation^39^. The contrasting proline responses observed here, with increased levels in *I. balsamina* and decreased levels in *B. oleracea* var. *acephala*, suggest differential stress coping strategies. Elevated proline in *I. balsamina* likely reflects a compensatory response to Pb-induced stress, whereas the reduction in *B. oleracea* var. *acephala* may indicate more efficient detoxification mechanisms that reduce the need for proline accumulation.

Similarly, total soluble protein (TSP) responses were strongly species-dependent. Pb exposure caused a dose-dependent decrease in TSP in *I. balsamina*, while *B. oleracea* var. *acephala* exhibited increased TSP levels, consistent with enhanced protein stability under stress. Such contrasting patterns have been reported in other species and cultivars, underscoring the role of genetic background in determining protein responses to Pb toxicity^24,40^. Together with proline dynamics, these protein-level changes indicate fundamentally different stress mitigation strategies between the two species.

Pb-induced protein damage activates cellular protein quality-control systems, including heat shock proteins (HSPs), which play a critical role in maintaining proteostasis under stress^41,13^. To our knowledge, this study provides the first comparative analysis of HSP60 and HSP23 responses to Pb stress in *I. balsamina* and *B. oleracea* var. *acephala*. In *I. balsamina*, HSP60 induction at lower Pb levels followed by a decline at higher concentrations suggests an early but limited protective response. In contrast, the progressive upregulation of HSP60 in *B. oleracea* var. *acephala* indicates a sustained capacity for protein stabilization under increasing Pb stress. Similarly, the strong induction of HSP23 in *B. oleracea* var. *acephala*, compared with its decline in *I. balsamina*, highlights divergent molecular strategies underlying Pb tolerance. Collectively, these molecular responses reinforce the superior Pb tolerance of *B. oleracea* var. *acephala* and support its potential application in Pb-contaminated environments.

## Conclusions

This study demonstrates that *I.* and *B. oleracea* var. *acephala* adopt distinct physiological, biochemical, and molecular strategies in response to Pb stress, providing the first comparative evidence of HSP60 and HSP23 responses in these species. Despite Pb-induced alterations in nutrient uptake and cellular processes, both species exhibited a considerable degree of tolerance, as reflected by stable chlorophyll content, limited membrane injury, and the absence of severe growth inhibition.

*B. oleracea* var. *acephala* displayed a stronger Pb tolerance characterized by effective root Pb retention, maintenance of protein homeostasis, and sustained induction of HSPs, supporting its potential suitability for phytostabilization of Pb-contaminated soils. In contrast, *I. balsamina* showed relatively higher Pb translocation to shoots together with enhanced proline accumulation, indicating a more responsive stress-adjustment strategy rather than long-term Pb immobilization.

Overall, these findings highlight species-specific adaptive mechanisms and emphasize the importance of integrating physiological, biochemical, and HSP-mediated molecular responses when assessing plant tolerance to Pb stress and their potential application in phytoremediation strategies.

## Materials and methods

### Plant material and lead treatments

Seeds of *Impatiens balsamina* L. ‘Pink Single’ and *Brassica oleracea* var. *acephala* ‘Nagoya Purple’ were purchased from a commercial seed supplier. The seeds of both species were sown simultaneously into peat-filled seedling trays. Seedlings of both species were grown for approximately three weeks under controlled greenhouse conditions before transplanting and irrigated regularly with tap water until healthy seedlings were obtained.

After seedling establishment, uniform plants were transplanted into 17 × 18 cm pots containing a peat:soil:perlite mixture (1:1:1), and the pot size was selected based on common greenhouse practices for ornamental plants to allow adequate root development without physical restriction during the experimental period. Growth medium characteristics are presented in Table 1. After transplanting, plants were allowed to acclimate and grow for an additional three weeks under controlled greenhouse conditions (22/18 °C day/night; 65% humidity) and natural greenhouse light before the initiation of Pb treatments. Thus, the total growth period from sowing to the initiation of Pb treatments was approximately six weeks. At the end of this period, Pb stress was imposed by irrigating plants with lead nitrate solutions at concentrations of 100, 200, and 400 ppm for six weeks^42^, while control plants received tap water. Throughout the experiment, plants were irrigated to slight drainage, and soil moisture was subsequently maintained at approximately 80% saturation. All plants were fertilized weekly with NPK (5:5:5) fertilizer (RootStar). For Pb-treated plants, the fertilizer was mixed into the lead nitrate irrigation solution, whereas control plants received the same fertilizer mixed with tap water. The same fertilization regime was applied to all treatments to ensure uniform nutritional conditions throughout the experiment. All plant-related experiments were conducted in accordance with institutional, national, and international guidelines and legislation.

### Determination of Pb and mineral nutrient concentrations in plants and growth medium

Lead (Pb) concentrations were determined in both plant tissues and growth medium samples. For plant analysis, harvested plant samples were dried at 70 °C for 72 h, ground into fine powder, and digested using a microwave digestion system (MARSXpress). Digestion was performed with 2 mL of 35% H₂O₂ and 5 mL of 65% HNO₃. Pb and mineral nutrient concentrations in the resulting digests were analysed using inductively coupled plasma optical emission spectrometry (ICP-OES; PerkinElmer Optima 8000), following the method described by Bouziani et al.^9^.

At the end of the experiment, growth medium samples from both control and Pb-treated pots were collected (5 g), dried at 70 °C for 72 h, powdered, sieved, and stored for analysis. Subsamples (500 mg) were digested with aqua regia (HCl:HNO₃, 3:1) using a microwave digestion system, filtered, and diluted to a final volume of 25 mL with deionized water. Pb concentrations in growth medium digests were subsequently determined using the same ICP-OES instrument, and Pb accumulation potential was calculated as described by Chandrasekhar and Ray^43^.

The bioenrichment factor (BCF) was calculated to assess the ability of plants to accumulate Pb from the growth medium. BCF values were determined separately for roots and shoots according to the method described by Cai et al.^44^ using the following equation (Eq. 1):

1 $$BCFroot or shoot=\frac{Pb concentration in root or shoot }{ Pb concentration in soil}$$

where Pb concentrations are expressed on a dry weight basis (mg kg⁻^1^). BCF values greater than 1 indicate effective Pb accumulation relative to the soil concentration, whereas values below 1 reflect limited enrichment capacity. In the present study, BCF was used as an indicator of Pb accumulation efficiency and comparative uptake behaviour between species rather than as a direct measure of field-scale phytoremediation performance.

### Physiological parameters

To assess injury, three 1.5-cm leaf discs from different plants were rinsed with deionized water and placed individually in test tubes containing 15 mL deionized water. Samples were shaken at 100 rpm for 4 h at room temperature, and initial conductivity (EC_initial_) was measured (Mettler Toledo, SevenEasy S30, Colombus Ohio, USA). For maximum conductivity (ECtotal), tubes were autoclaved at 121 °C for 15 min, cooled to room temperature, and remeasured. Ion leakage was calculated using Eq. (2). and injury was estimated according to Ilík et al.^27^, using Eq. (3):

2 $$Ion leakage\left(\%\right)=\frac{ECinitial}{ECtotal}x100$$

3 $$Injury\left(\%\right)=L\left(t\right)\%-L\left(c\right)\% /\left(100-L\left(c\right)\%\right) x100$$

where L(t) represents the treatment’s ion leakage percentage and L(c) the control’s ion leakage percentage.

Leaf relative water content (RWC, %) and turgor loss were determined following Gulen and Eris^45^. Three 1.5-cm leaf discs were collected in three replicates per treatment. Fresh weight (Fw) was recorded, turgor weight (Tw) was measured after 4 h in deionized water, and dry weight (Dw) after drying at 70 °C for 24 h. RWC and turgor loss were calculated using Eq. (4) and (5).

4 $$\mathrm{RWC}\left(\mathrm{\%}\right)=\frac{\mathrm{Fw}-\mathrm{Dw}}{\mathrm{Tw}-\mathrm{Dw}}\mathrm{x}100$$

5 $$\text{Loss of turgidity}\left(\mathrm{\%}\right)=\frac{\mathrm{Tw}-\mathrm{Fw}}{\mathrm{Tw}}\mathrm{x}100$$

From fully developed leaves of each treatment, three 0.5-cm discs were incubated in 5 mL DMF for 72 h at 4 °C in the dark. Chlorophyll content was then measured at 652 nm using a spectrophotometer (Perkin Elmer Lambda 25 UV/VIS, USA) and calculated according to Moran and Porath^46^.

### Plant biomass

At the end of the experiment, three plants per treatment were collected in triplicate. Shoots and roots were separated, roots were washed and blotted dry, and fresh weights were recorded. Samples were then dried at 70°C to constant weight, and dry weights were determined^47^.

### Proline content and protein analysis

For proline analysis, 0.2 g of leaf tissue was homogenized in 3% sulfosalicylic acid and centrifuged at 5000 × g for 15 min at 4°C^48^. The supernatant was reacted with acid ninhydrin and acetic acid at 100°C for 1 h, cooled, extracted with toluene, and absorbance was measured at 520 nm. Proline concentration was calculated using a standard curve.

For total soluble protein (TSP) extraction, 0.25 g leaf tissue was homogenized in cold borate buffer and centrifuged at 10,000 × g for 10 min at 4°C. TSP content was determined by the Bradford^49^ method. Equal amounts of protein (10 μg) were separated via SDS-PAGE, stained with Coomassie dye, and used for immunoblotting with HSP23 (Sigma) and HSP60 (Sigma) antibodies (1:1500). Blots were visualized using the ProtoBlot Western Blot AP Kit (Promega), and band intensities were quantified with NIH Image software (2024). Relative HSP levels were quantified by normalizing band intensities to those of the corresponding control groups, which were set at 100%.

### Statistical analysis

Each trial was repeated three times. The obtained data were determined by Duncan’s test at *p* ≤ 0.05. Statistical analyses were performed with SPSS for Windows software.

## Supplementary Information

Supplementary Information 1

## Data Availability

All data generated or analysed during this study are included in this published article (and its Supplementary Information files).
