# Supplementary material for: Differential physiological, nutritional and molecular responses to lead stress in garden balsam and ornamental kale
Source: Sci Rep. 2026 Apr 3;16:16054. doi: 10.1038/s41598-026-46310-6 (PMC13199446; doi:10.1038/s41598-026-46310-6)
Supplement: Supplementary file 1 — Supplementary Information 1 [file 41598_2026_46310_MOESM1_ESM.pdf]

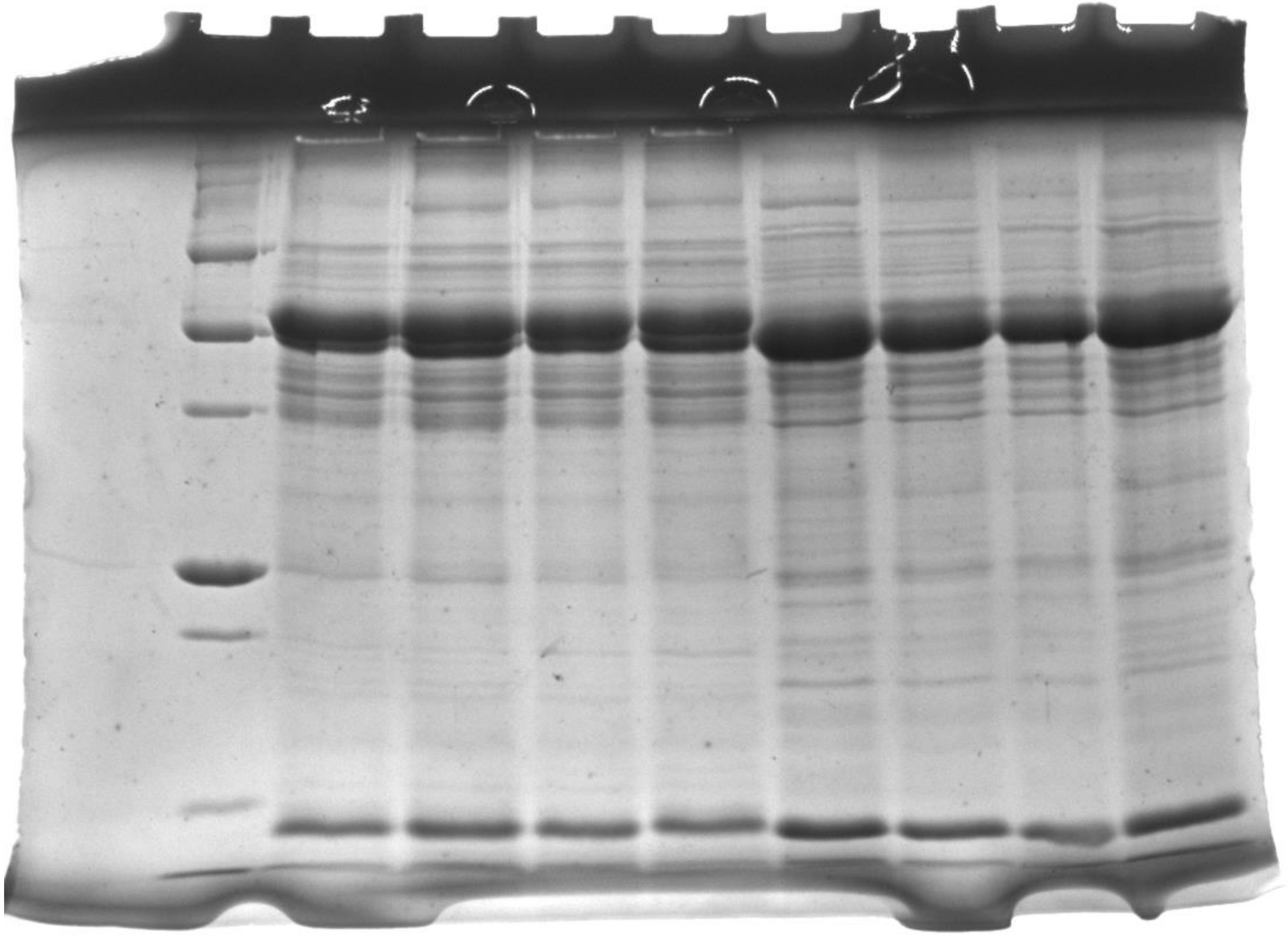

Figure 3A: SDS-PAGE profiles of total soluble proteins in *I. balsamina* and *B. oleracea* var. *acephala*.

M: Marker
Lane 1: *I. balsamina* control
Lane 2: *I. balsamina* 100 ppm
Lane 3: *I. balsamina 2*00 ppm
Lane 4: *I. balsamina 4*00 ppm
Lane 5: B. *oleracea* control
Lane 6: B. *oleracea* 100 ppm
Lane 7: B. *oleracea 2*00 ppm
Lane 8: B. *oleracea 4*00 ppm

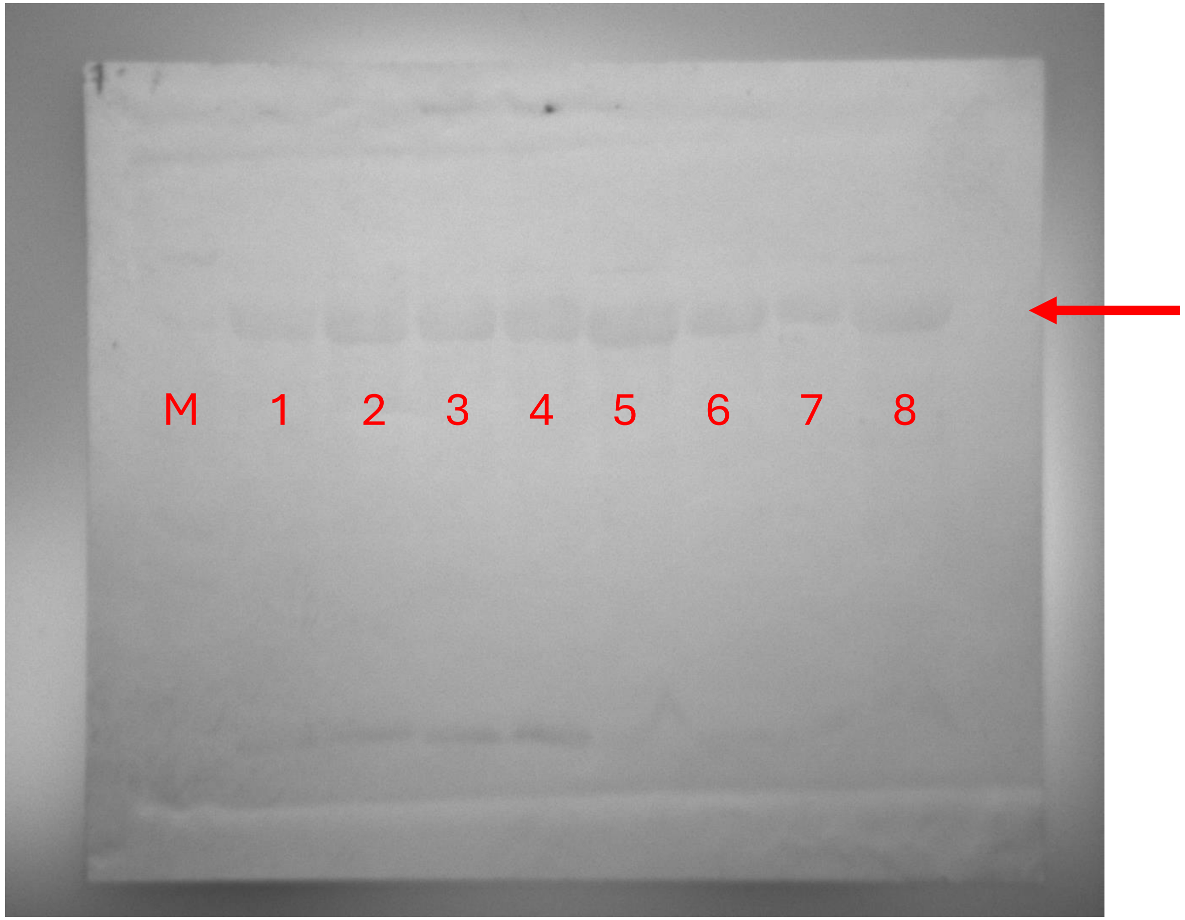

Figure 3B: Immunoblot detection of HSP60. The red arrow indicates the relevant protein band.

M: Marker
Lane 1: *I. balsamina* control
Lane 2: *I. balsamina* 100 ppm
Lane 3: *I. balsamina* 200 ppm
Lane 4: *I. balsamina* 400 ppm
Lane 5: B. *oleracea* control
Lane 6: B. *oleracea* 100 ppm
Lane 7: B. *oleracea* 200 ppm
Lane 8: B. *oleracea* 400 ppm

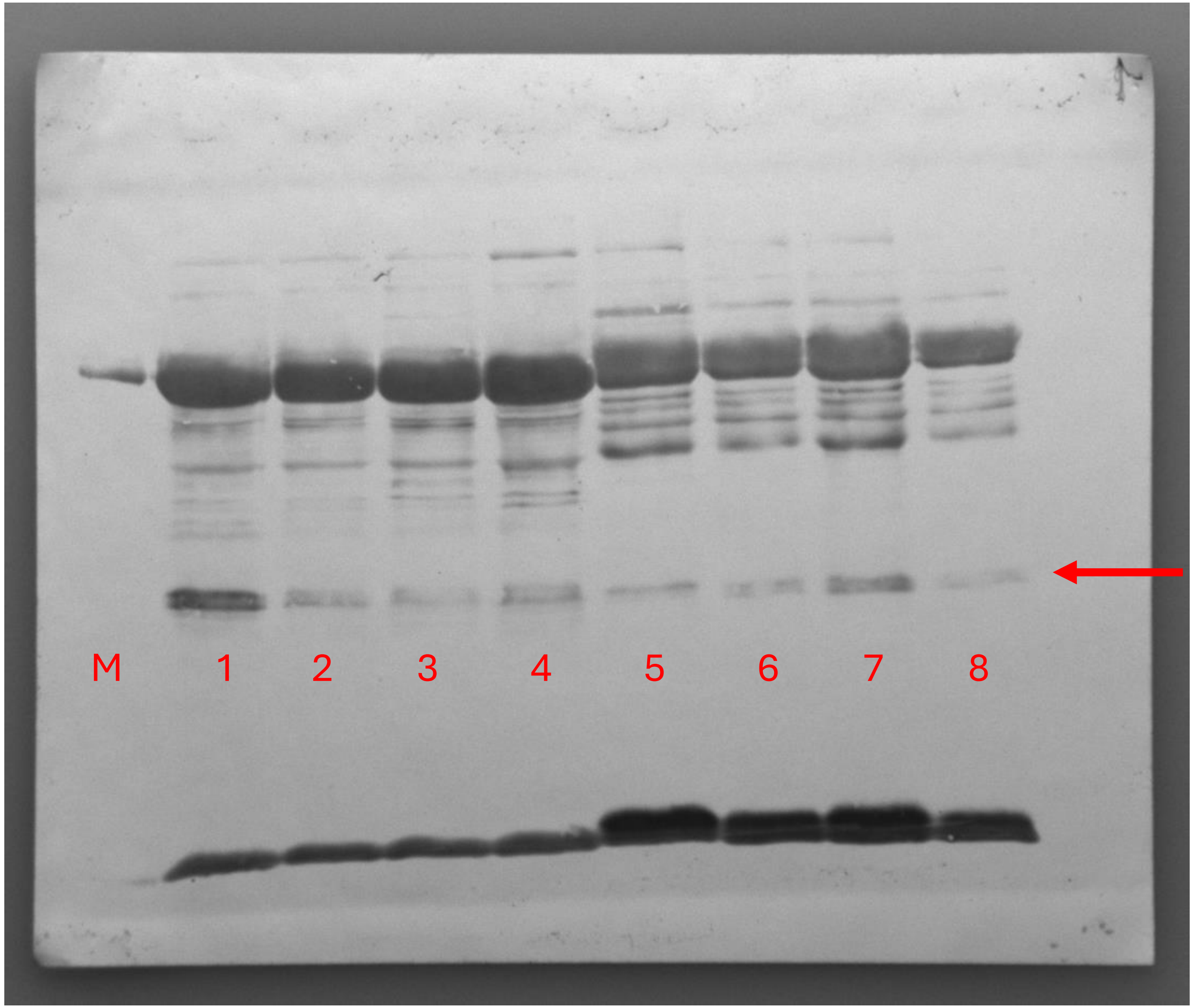

Figure 3C: Immunoblot detection of HSP23. The red arrow indicates the relevant protein band.

M: Marker
Lane 1: *I. balsamina* control
Lane 2: *I. balsamina* 100 ppm
Lane 3: *I. balsamina* 200 ppm
Lane 4: *I. balsamina* 400 ppm
Lane 5: B. *oleracea* control
Lane 6: B. *oleracea* 100 ppm
Lane 7: B. *oleracea* 200 ppm
Lane 8: B. *oleracea* 400 ppm
